# Arthropod-Borne Pathogens in Stray Cats from Northern Italy: A Serological and Molecular Survey

**DOI:** 10.3390/ani10122334

**Published:** 2020-12-08

**Authors:** Valentina Virginia Ebani, Lisa Guardone, Federica Marra, Iolanda Altomonte, Simona Nardoni, Francesca Mancianti

**Affiliations:** Department of Veterinary Sciences, University of Pisa, Viale delle Piagge 2, 56124 Pisa, Italy; valentina.virginia.ebani@unipi.it (V.V.E.); vet.federicamarra@gmail.com (F.M.); altomonte@vet.unipi.it (I.A.); simona.nardoni@unipi.it (S.N.); francesca.mancianti@unipi.it (F.M.)

**Keywords:** vector-borne, PCR, seroprevalence

## Abstract

**Simple Summary:**

Monitoring the health status of cats ensures their welfare and control of infections transmissible to other domestic and wild animals, as well as to humans. In the present survey, blood samples and ticks were collected, between February 2018 and October 2019, from 85 stray cats living in registered colonies in Emilia Romagna (northern Italy), with the aim of investigating the presence and infection level of a wide range of pathogens transmitted by arthropods (arthropod-borne pathogens—ABPs). The collected samples were subjected to serological and DNA-based methods. The presence of pathogens was observed in 71 cats (83.5%) using serological methods and in 47 cats (55.3%) by applying DNA-based analysis. Coinfections (presence of two or more pathogens) were observed in 21 cats (24.7%). While ABPs in privately owned cats are more easily prevented and managed through standard veterinary care, stray cats may be particularly at risk as they live outdoors, have constant exposure to arthropods such as ticks and fleas, and generally do not receive regular antiparasitic treatment. Indeed, the results of the present study show a widespread presence of ABPs, suggesting that stray cats may represent a potential health threat to companion animals and people and the need for improved management.

**Abstract:**

Cats may be affected by a wide range of arthropod-borne pathogens (ABPs) of medical and veterinary interest. Between February 2018 and October 2019, 85 blood samples were collected from stray cats from the Emilia Romagna region (northern Italy). Ticks (*n* = 28) on the examined cats were also collected. Serological and molecular methods were applied to search for infection by *Anaplasma phagocytophilum*, *Bartonella henselae*, *Coxiella burnetii*, *Ehrlichia canis*, *Leishmania* spp., *Babesia* spp., *Hepatozoon* spp., and *Cytauxzoon* spp. A total of 71 sera (83.5%) had antibodies to at least one investigated pathogen: 39 (45.9%) were positive for *B. henselae*, 32 (37.6%) positive for *C. burnetii*, 12 (14.1%) positive for *E. canis*, four (4.7%) positive for *A. phagocytophilum*, and two (2.4%) positive for *Leishmania* spp. A total of 47 (55.3%) DNA samples were positive by PCR for at least one investigated pathogen: 25 (29.4%) were positive for *C. burnetii*, 23 (27.1%) positive for *B. henselae*, two (2.4%) positive for *E. canis*, five (5.9%) positive for *Leishmania* spp., and two (2.4%) positive for *Cytauxzoon* spp. Coinfections were observed in 21 cats (24.7%). No positivity was found for *A. phagocytophilum*, *Babesia* spp., or *Hepatozoon* spp. All ticks were negative. A widespread presence of ABPs in the investigated area of northern Italy was shown. Accurate information on their prevalence may be relevant for feline veterinary medicine, as well as from a One Health perspective.

## 1. Introduction

Domestic cats (*Felis catus*) may host a wide range of protozoa and bacteria of medical and veterinary interest [1,2,3,4,5,6], and they are often exposed to hematophagous arthropods, including sand flies, fleas, and ticks [7]. Infections by different arthropod-borne pathogens (ABPs) have been investigated in feline populations, even though less frequently than in dogs [7,8]. In fact, epidemiological data related to the spreading of ABPs among pets, in Europe and in the rest of the world, have been performed mainly in dogs [9,10]. Moreover, whereas laboratory diagnosis for arthropod-borne infections on the basis of clinical signs is often requested for this latter species, these infections are not frequently diagnosed and, thus, underestimated in feline populations [7]. Arthropod-borne diseases, caused by ABPs, have emerged or re-emerged in the past few years due to human factors and climatic changes [11]. Thus, improved knowledge of various aspects of these infections in feline hosts, such as occurrence and distribution in different geographic areas, is required [8,12].

Among the most relevant protozoan ABPs are feline leishmaniosis, babesiosios, cytauxzoonosis, and hepatozoonosis. Feline leishmaniosis (FeL), caused by *Leishmania infantum*, is reported from areas endemic for canine leishmaniosis [4,13]. Cat infection is frequent and clinical signs occur mostly as nodular skin lesions; when these appear, specific treatment is needed, although cats frequently do not fully recover. These animals have been recognized able to infect phlebotomine vectors, playing a role as minor reservoirs [14]. Feline babesiosis is considered rare in Europe [15]. Two clinical cases were reported from Germany and Poland, caused by a large *Babesia* morphologically very similar to *B. canis*. The affected animals showed fever, anemia, and hematuria and responded to treatment with imidocarb [16,17]. In general, cats may be infected by several *Babesia* spp., recently reviewed by Penzhorn and Oosthuizen [15]. Cytauxzoonosis, an emerging tick-borne protozoan disease affecting domestic and wild felids, is caused by piroplasms of the genus *Cytauxzoon*, specifically *C. felis*, *C. manul*, and *Cytauxzoon* sp. [18]. The most common clinical features are fever, anorexia, lethargy, and icterus [19]. *C. felis*, which is limited to the American continent (United States and Brazil) [20,21], was previously considered responsible for life-threatening infections leading to the death of domestic cats within a few days [22]. However, a different pathogenicity, depending on the strains, was recently reported [22], and even domestic cats developing subclinical and persistent blood parasitaemia have been described [23,24,25], suggesting they may play a role as reservoir of infection [25]. Fewer epidemiological and clinical data on infections by species other than *C. felis* are available, especially for Europe [18,26], although several studies have been published lately [27,28,29,30]. Finally, *Hepatozoon felis*, *H. canis*, and *H. silvestris* are the etiological agents of feline hepatozoonosis [2,31,32]. The infection is primarily transmitted by the ingestion of infected ticks. Hepatozoonosis in cats is usually asymptomatic but may develop as a muscle disease [27].

Among the bacterial ABPs, *Bartonella henselae* is the most known agent affecting cats. Other relevant bacteria species are *Coxiella burnetii*, *Anaplasma phagocytophilum*, and *Ehrlichia canis.* The *Bartonella* genus includes several species able to infect domestic and wild animals. These bacteria are transmitted by blood-sucking arthropods, including fleas, lice, and sandflies, whereas it is not clear if ticks may be considered as potential vectors [33]. In particular, *B. henselae* is transmitted by the flea *Ctenocephalides felis* and is associated with cat scratch disease (CSD) in humans [34]. *Coxiella burnetii* is an obligate intracellular Gram-negative bacterium, which causes a zoonosis called Q Fever. Several domestic and wild animal species are known to be susceptible to this agent, even though Q Fever is usually related to cattle and small ruminants. Coxiellosis-associated abortion has been documented in cats; however, the organism has also been isolated from cats after normal parturition [35,36]. *Anaplasma phagocytophilum* and *E. canis* are two obligate intracellular Gram-negative bacteria responsible for well-known canine diseases. They are mainly transmitted by *Ixodes ricinus* and *Rhipicephalus sanguineus* ticks, respectively. Clinical signs and abnormal laboratory findings related to feline ehrlichiosis and anaplasmosis are similar to those observed in dogs, and include anorexia, hyperesthesia, lethargy, weight loss, joint pain, dyspnea, lymphadenomegaly, anemia, and hyperglobulinemia [37,38].

All these diseases should not be overlooked, as, for instance, feline leishmaniosis is increasingly recognized as a disease of cats in endemic areas [4,13]. Many surveys conducted in cats examined only one parasitic agent, but studies should ideally examine several pathogens [39]. In particular, studies on stray colony cats are particularly valuable for assessing the epidemiology and emergence of ABPs, as they generally do not receive regular antiparasitic treatment [5,6,12,40]. Thus, this survey aimed to investigate the occurrence of bacterial ABPs, specifically *A. phagocytophilum*, *B. henselae*, *C. burnetii*, and *E. canis*, and protozoan ABPs, including *Leishmania* spp., *Babesia* spp., *Cytauxzoon* spp., and *Hepatozoon* spp., in stray cats from northern Italy (Emilia Romagna region) and in ticks collected on the same animals.

## 2. Materials and Methods

### 2.1. Sampling

Between February 2018 and October 2019, 85 venous blood samples were collected from stray cats (37 females and 48 males), during ovariohysterectomy and orchiectomy activities conducted by veterinarians of the Italian National Health Service (local unit of Bologna). All stray cats belonged to registered colonies of the province of Bologna and Rimini (Emilia Romagna region, northern Italy). No sex, age, or clinical condition inclusion criteria was applied. Body condition score (BCS) was evaluated (score 1–9) [41]. Blood samples were collected in EDTA (ethylenediaminetetraacetic acid) and serology tubes from the jugular vein after trichotomy and disinfection of the neck region with ethyl alcohol, with the animal under general anesthesia. Serum was collected from clotted blood samples after centrifugation at 1200× *g* for 10 min.

Ticks occurring on the examined cats were removed, placed in 1.5 mL tubes with 70% ethanol. Blood, serum, and tick samples were then transferred to the Department of Veterinary Sciences of the University of Pisa for the analytical procedures. Herein, the ticks were morphologically identified [42]. Serum, blood, and tick samples were stored at −20 °C until used.

### 2.2. Serological Analysis

#### 2.2.1. Indirect Immunofluorescence Assay for Bacterial Pathogens

An indirect immunofluorescent assay (IFA) was carried out to detect antibodies against the investigated bacterial pathogens. Commercial IFA slides (Fuller Laboratories, Fullerton, CA, USA) with the following coated antigens were used: *A. phagocytophilum*, *B. henselae*, *C. burnetii* (separate phase I and phase II antigens), and *E. canis*. A commercial fluorescein isothiocyanate-conjugated sheep anti-cat immunoglobulin G (IgG) (Sigma-Aldrich, St. Louis, MO, USA) diluted 1:50 in Evans Blue (Sigma-Aldrich) solution was used as secondary antibody.

Antibody titers of 1:40 and 1:64 were considered the cut-off values for *A. phagocytophilum*/*E. canis* and *B. henselae*/*C. burnetii*, respectively [43,44,45]. Two-fold dilutions of the positive sera were tested to determine the endpoint titer.

#### 2.2.2. Indirect Immunofluorescence Assay for *Leishmania*

An IFA test was applied for the detection of antibodies against *Leishmania* spp., as described by Mancianti and Meciani [46], using cultured promastigotes fixed on Multiwell glass slides as antigens. Sera were tested starting from a 1:10 dilution.

### 2.3. Molecular Analysis

#### 2.3.1. DNA Extraction

Total DNA was extracted from the sediment (200 µL) obtained after centrifugation of the blood samples and from ticks using the DNeasy Blood & Tissue Kit (Qiagen, Milano, Italy), following the manufacturer’s blood and tissues protocols, respectively. Each DNA sample was stored at −20 °C until used in PCR assays.

#### 2.3.2. DNA Amplification and Sequencing

Different PCR approaches were employed to detect the investigated pathogens in DNA extracted from blood and ticks, following the protocols previously described and summarized in Table 1. For some pathogens, such as *Leishmania* spp., *A. phagocytophilum*, and *E. canis*, a nested PCR protocol was used. All DNA samples resulted positive for *Bartonella* spp. were successively tested with a PCR protocol to identify *B. henselae* and distinguish type I and type II, using reverse type-specific primers BH1 or BH2 in combination with the forward broad-host-range primer 16SF [47]. In addition, DNA extracted from ticks was used for specific identification of the host, targeting a fragment of the mitochondrial 16S ribosomal DNA (rDNA) gene [48].

PCR assays were performed with Wonder Taq (EUROCLONE, Italy) in an automated thermal cycler (Gene-Amp PCR System 2700, Perkin Elmer, Norwalk, CT, USA). Sterile distilled water was used instead of DNA in the negative control. DNA extracted from slides used for indirect immunofluorescent assay coated with *A. phagocytophilum*, *E. canis*, *C. burnetii*, and *B. henselae* (Fuller Laboratories, Fullerton, CA, USA) and known positive samples, previously sequenced, of *B. annae* [49], *Cytauxzoon* sp. [18], *H. canis* [49], and *L. infantum* were included as positive controls [50].

PCR products were analyzed by electrophoresis on 2% agarose gel stained with GelRed^®^ Nucleic Acid Gel Stain (Biotium). SharpMass™ 100 Plus Ladder (Euroclone, Milano, Italy) was used as a DNA marker and for visual estimation of the PCR products’ concentration.

For *Cytauxzoon* spp., PCR products of the expected length and with a sufficient concentration were forward and reverse Sanger sequenced by an external company. Nucleotide sequences were analyzed using Bioedit version 7.0.9 [51]. Adjustments were made after visual checking and consensus sequences were compared against those deposited in GenBank by using the National Center for Biotechnology Information (NCBI) Basic Local Alignment Search Tool (BLAST). A neighbor-joining (NJ) phylogram was constructed using Bioedit and MEGA-X software including the DNA sequences obtained in the present study and other sequences of *Cytauxzoon* spp. previously deposited in GenBank [18,26,28,30,52,53,54,55,56,57]. Sequencing and phylogenetic analysis was conducted only on *Cytauxzoon* spp. to determine the species involved and as reports of this parasite in Italy are still quite uncommon.

### 2.4. Statistical Analysis

The detection rate was calculated for each pathogen and each test, together with the 95% confidence interval (CI).

## 3. Results

The overall results of the serological and molecular analysis are reported in Table 2. Briefly, the highest seropositivity rate was observed for *B. henselae* (45.9%), followed by *C. burnetii* (37.6%), *E. canis* (14.1%), *A. phagocytophilum* (4.7%), and *Leishmania* sp. (2.4%). The detection rate found by PCR were generally lower; all cats positive by PCR to the bacterial pathogens were also seropositive, while three cats PCR-positive to *Leishmania* were seronegative (see Section 4). Coinfections by two or more pathogens were observed in 21 cats (24.7%) (Table 3). Overall, the BCS most frequently scored 5, whereas only a minority of cats scored 7 (5.9%) or 3 (3.5%).

### 3.1. Serological Analysis

A total of 71 (83.5%; 95% CI 75.6–91.4%) sera had antibodies to at least one investigated pathogen. With regard to bacterial pathogens, 39 cats (45.9%; 95% CI 35.3–56.5) were positive for *B. henselae* (*n* = 18 titer 1:64, *n* = 21 titer ≥1:128), 32 cats (37.6%; 95% CI 27.3–47.9) for *C. burnetii* (phase II antigen, *n* = 14 titer 1:64, *n* = 18 titer ≥1:1.128), 12 cats (14.1%; 95% CI 6.7–21.5) for *E. canis* (*n* = 9 titer 1:40, *n* = 3 titer ≥1:80), and four cats (4.7%; 95% CI 2–9.2%) for *A. phagocytophilum* (titer 1:40). Serological tests for protozoan showed that two (2.4%, 95% CI 0–5.6%) of the sampled cats were positive for *Leishmania* sp. with a titer 1/10.

### 3.2. Molecular Analysis

A total of 47 (55.3%; 95% CI 44.7–65.9%) DNA samples were positive by PCR for at least one investigated pathogen. Among these, 25 (29.4%; 95% CI 19.7–39.1%) cats were positive for *C. burnetii*, 23 (27.1%; 95% CI 17.6–36.5%) for *B. henselae* (16 genotype I and 7 genotype II%), and two (2.4%; 95% CI 0–5.6%) for *E. canis*. No cats were positive at PCR for *A. phagocytophilum*.

The molecular analysis confirmed positivity for *Leishmania* in the two seropositive cats, and DNA of this parasite was also found in three additional cats (overall prevalence 5.9%; 95% CI 0.9–10.9%). Moreover, DNA of *Cytauxzoon* sp. (confirmed by sequencing) was found in two cats (2.4%; 95% CI 0–5.6%), while no positivity was found for *Babesia* spp. or *Hepatozoon* spp.

**Figure 1:**
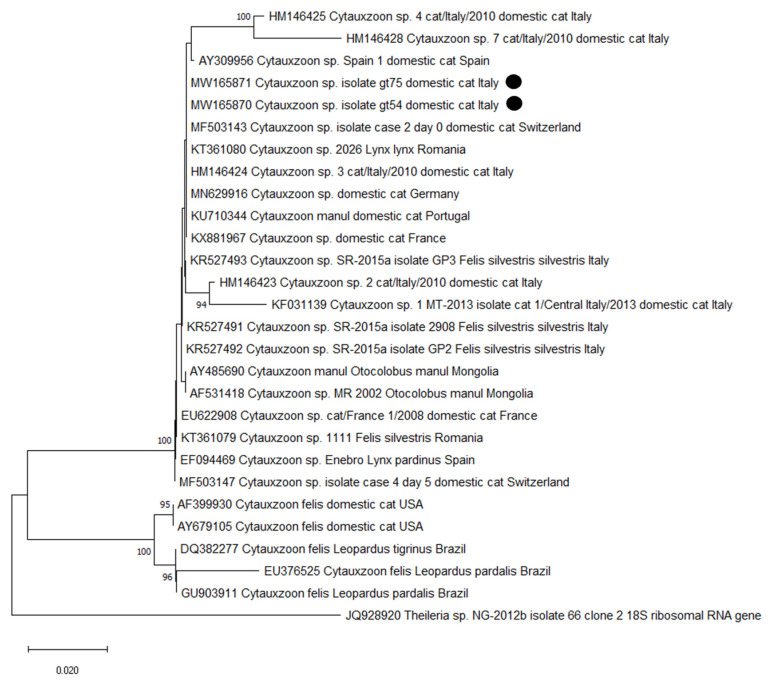
Neighbor-joining phylogram created with the two sequences of the small subunit (18S) ribosomal RNA gene obtained in the present survey (highlighted with a black dot), 20 sequences of *Cytauxzoon* sp., and five sequences of *Cytauxzoon felis* from other studies in domestic and wild animals.

The two sequences of *Cytauxzoon* sp. were deposited in GenBank (MW165870-MW165871). The BLAST analysis retrieved 100–99.75% identity with sequences deposited as *Cytauxzoon* sp. isolated from domestic cats in Germany (MN629916 [30]), in Switzerland (MF503141-8 [29]; KU306940-4, KU306945-8, Willi et al., Tochtermann et al. unpublished), in France (KX881967 [28]; EU622908 [52]), and in Portugal (KU710344, [26]), as well as with isolates from the European lynx and the wild cat in Romania (KT361070-2, KT361074, KT361076, KT361079-81 [53]) and from the Iberian lynx in Spain (EF094468-70 [54]). Accordingly, in the NJ dendrogram the sequences obtained in this study clustered with other *Cytauxzoon* sp. isolates obtained from wild and domestic felids from Italy, Spain, France, Switzerland, Germany, Portugal, and Romania and with *C. manul* found in Pallas’s cats (*Otocolobus manul*) from Mongolia [55,66]. As already observed in other studies [18,26,56], isolates of *C. felis* from the American continent belong to a different cluster (Figure 1).

The majority of the 28 ticks were molecularly identified as *I. ricinus* (92.8%), followed by *R. sanguineus* (7.2%). No pathogen DNA was found in ticks.

## 4. Discussion

Data obtained in the present investigation show that ABPs are widespread among stray cats from the investigated geographic areas. Feline ABP occurrence and distribution among Italian cat populations are described in many studies, mainly focusing on southern regions and insular areas [4,8,67,68,69,70], while northern [5,6,56] and central Italy had since recently received little attention [7,43,44]. A large survey investigating owned cats from northern, central, and southern Italy was also recently published [71].

The high percentage of cats that resulted PCR-positive to *C. burnetii* is worthy of attention. This pathogen is traditionally related to farm animals, mainly ruminants, in which it causes reproductive disorders. The presence of a large number of bacteria mainly in placenta, birth fluids, and milk causes a high risk of infection for workers having contact with infected animals and their products. Moreover, raw milk and unpasteurized dairy products may be cause of infection for consumers. *C. burnetii* is able to infect other mammals, and infections in cats have been previously reported. Cases of abortion have been described, although the organism has also been isolated from cats after normal parturition [35,36]. Data about the spreading of *C. burnetii* in domestic and stray feline populations are scant, but information to better understand the role of cats in the epidemiology of Q fever is necessary in view of the One Health concept. Whereas, according to some authors who did not find *C. burnetii* DNA in cats’ samples, cats are not important reservoirs for human Q Fever infection [72], our results, with a 29.4% of bacteremic animals, suggest that cats are involved in the epidemiology of coxiellosis and could be source of infection for other animals and people. Owners, as well as veterinarians, breeders, and other persons having contact with cats, may be at risk of infection mainly with the abortion products; however, urine, feces, and respiratory secretions could also contain coxiellae. Other than the transmission through oral and inhalatory routes, *C. burnetii* may be transmitted by tick bites [73]. During our study, *C. burnetii* DNA was not found in the analyzed ticks. Positive reactions to the agent found in the tested cats could be related to the ingestion of infected roditors and/or contact with them, which is more frequent in stray cats. In fact, rodents are considered natural reservoirs of this pathogen [74]. Among the examined cats, 14.1% had antibodies against *E. canis*, and, in 2.4%, DNA of the pathogen was found. Overall, 4.7% of the cats were seropositive for *A. phagocytophilum*, even though all them were PCR-negative, indicating exposure to the pathogen but not active infection. While, for the Emilia Romagna region, data are scanty, a previous serological survey in cats from the neighbouring region Tuscany found seroprevalence values of 6.4% and 4.5% to *E. canis* and *A. phagocytophilum*, respectively [44]. The lower prevalence to *E. canis* detected in the previous survey could be related to the better life condition of the cats that lived in private catteries and domestic environments. On the other hand, the molecular prevalence values detected in this study were lower than those observed among stray cats from urban colonies in Milan (northern Italy). In fact, Spada et al. [5] found 17.7% of PCR-positive cats for *A. phagocytophilum* and 5.4% for *Ehrlichia* spp. On the contrary, no DNA of *Ehrlichia/Anaplasma* spp. was found in another recent study including cats from throughout Italy [71]. *E. canis* and *A. phagocytophilum* are usually transmitted to dogs by *R. sanguineus*, *Ixodes* spp., *Amblyomma* spp., and *Dermacentor* spp. ticks [75]. It has been supposed that these pathogens are also transmitted by ticks to cats, even though the transmission mechanism in these animals is actually not fully understood. In the present study, DNAs of the two pathogens were not found in the examined ticks. Cats could contract *E. canis* and/or *A. phagocytophilum* from other ticks that previously fed on them or from other hematophagous arthropods. *Ctenocephalides felis* fleas, which are proven vectors of other feline pathogens and infest cats more frequently than ticks, should also be investigated to determine if they are able to transmit *E. canis* and *A. phagocytophilum* [44].

The present survey found a high seroprevalence (45.9%) to *B. henselae*. Moreover, 27.1% of the tested cats had *B. henselae* DNA in their blood. Both detected percentages were higher than those reported in a previous investigation carried out in domestic cats that lived in private houses and/or gardens [43]. Our prevalence value is higher than the value found by Latrofa and collaborators [71], where 24 out of 958 (2.5%) cats tested PCR-positive for *Bartonella* spp., with 1.6% for *B. henselae* and 0.9% for *B. clarridgeiae*. The present investigation, as well as the above-cited study, found a higher number of cats positive to *B. henselae* genotype I, suggesting that it is more widespread than genotype II. However, previous studies demonstrated that the prevalence of the two genotypes may vary in relation to the considered geographic area [76]. Cats are usually asymptomatic when infected with both genotype I and genotype II; conversely, it seems that the two types may induce varying pathologic features in infected people. In particular, genotype I is considered more pathogenic for humans than genotype II [77,78]. It is well known that *B. henselae* is transmitted by *C. felis*, whereas it is not clear if ticks may be considered as potential vectors. In fact, there is little evidence that *Bartonella* spp. can replicate within ticks and no definitive evidence of transmission by a tick to a vertebrate host [33]. The absence of *B. henselae* DNA in the examined ticks could confirm that these arthropods are not important in the epidemiology of the agent.

With regard to protozoan ABPs, the presence of *Leishmania* sp. is an interesting finding. Feline leishmaniosis (FeL) due to *L. infantum* was firstly described in Italy in a cat from Imperia (Liguria, Italy) [13], thus living in a highly endemic area for canine leishmaniosis. Thenceforth, along with the improvement of feline medicine and veterinary clinical diagnostic tools, the reports from Europe have become more and more frequent. The seroprevalence values range from more than 60% to 0% [79] depending on the canine seroprevalence range. Moreover, the positivity to specific parasitological tests is not necessarily related to an ability to develop an overt disease. Serological testing is considered to have a very low sensitivity, although it has shown 100% specificity [80]. It is believed that the immune response to *Leishmania* strongly differs between dogs and cats. The seroprevalence, as well as the antibody titers in epidemiological surveys, is lower than reported in dogs. Another basic issue impacting on the seroprevalence/PCR positivity estimation, such as for dogs, is the season of testing. In fact, in some cats, a transient disseminated infection would occur during the transmission period. The cats of the present study were sampled between October and June; thus, the positivity rate should not be overestimated. The finding of antibodies and/or parasite DNA would indicate that the animals were infected; nevertheless, the occurrence of DNA in seronegative cats can be explained with the very low humoral response in this animal species. Considering that, in the Bologna province, a stable focus of canine leishmaniosis has been reported [81], the finding of low prevalence of infection in cats is not surprising. In accordance, the spread of *L. infantum* infection in cats was also observed in a study on owned cats sampled at a veterinary university hospital in Bologna (P 12.5%) [82]. A slightly lower prevalence (8.6% considering PCR and IFA results combined) of FeL was recently found in Milan (northern Italy), a non-endemic area for this parasitic disease. Although it was not clear if these were imported cases or if *Leishmania* vectors are present in the area, the study showed a stable FeL situation among the stray cats of the city compared to previously available studies [83].

The finding of *Cytauxzoon* sp. in two cats confirms the presence of this pathogen in Italy, as already observed both in domestic cats [2,56,57] and in wild cats [18]. As mentioned, *C. felis*, the first described species of the genus, is distributed in the New World where, in addition to cases in domestic cats, it is traditionally associated with the wild felid *Lynx rufus* (bobcat), the reservoir in endemic areas [21,22]. A closely related piroplasm was reported in Pallas’s cats (*O. manul*) from Mongolia and described as a new species, *C. manul*, on the basis of a significant sequence divergence [55,66]. In Europe, cytauxzoonosis by *C. felis* has not been described; however, in the past decade, infections with a genetically distinct species described as *Cytauxzoon* sp. have been increasingly reported [28] in several European countries, both in domestic cat [26,27,28,29,30,56,57] and in wild felids, including the Iberian lynx (*L. pardinus*), the European lynx (*Lynx lynx*), and the European wild cat (*Felis silvestris silvestris*) [18,52,53,54,84,85]). The sequences described from Europe as *Cytauxzoon* sp. are closely related and may belong to the same species [28]. On the contrary, isolates of *C. felis* from the United States of America (USA) and from South America cluster separately from the European ones and from each other (Figure 1). The negligible differences between the 18S rDNA gene from European wild and domestic felids and the available sequences of *C. manul* suggest their conspecificity [26]. However, different genes with higher variability than 18S rRNA, such as the internal transcribed spacer1 (ITS1) and ITS2 [86] or the III subunit of the cytochrome oxidase [87], are needed to further clarify the taxonomy of the species of *Cytauxzoon* described until now [56]. Thus, life cycle and genetic markers need to be fully understood before referring the European *Cytauxzoon* isolates to *C. manul* [53].

Clinical cytauxzoonosis in domestic cats has been recently described in Germany, Switzerland, France, and Portugal. In Italy, following the detection of three clinical cases in cats from Trieste (northeastern Italy), an epidemiological study was carried out in colony (*n* = 63) and owned (*n* = 52) cats from the same city, finding *Cytauxzoon* sp. in 23% of the examined cats. A subsequent report of two other clinical cases from the same authors confirmed the presence of the pathogen in northeastern Italy, as well as showed its presence in central Italy [57]. On the contrary, this protozoan ABP was not detected in cats in other Italian surveys [5,7,70]. In the Italian cases, infection was mainly subclinical, as clinical disease was observed only in seven cats [56]. Thus, it has been suggested that *Cytauxzoon* sp. in Europe is less virulent than *C. felis* and that the disease could develop preferentially in the case of concurrent disease or immunodeficiency [28,56]. In general, such infections are likely to be underdiagnosed, and causes and impacts of feline cytauxzoonosis have not yet been clarified [7]. Furthermore, the epidemiology of the infection in this country is still not fully clear. The only study on wild felids in Italy found a prevalence of 14.3% [18], similar to the value detected in Iberian lynxes from Spain (15%) [54].

As for the arthropod host, ticks known to be able to transmit *C. felis* infection (mainly *A. americanum* and *Dermacentor varibilis*) are not described in Italy; thus, it is hypothesized that other Ixodid ticks commonly found in the country, i.e., *I. ricinus* or *Dermacentor* sp., might be involved in the transmission of *Cytauxzoon* sp. [56]. However, as in the present study, the arthropods collected in the focus of cytauxzoonosis in domestic cats of northeastern Italy (one *R. sanguineus* s. l., nine *I. ricinus*, and 28 fleas *C. felis*) were all negative by PCR, even when collected from five *Cytauxzoon*-positive cats [57]. In general, very low prevalence values for several pathogens (*A. phagocytophilum, B. henselae, Hepatozoon* sp.) were observed in a large-scale molecular study on ticks from cats in the United Kingdom (UK) [88].

The observed negativity for *Babesia* sp. agrees with other recent Italian surveys [7,71]. However, in other Italian studies, *B. microti* DNA was detected in cat blood samples [6], and a seropositivity rate around 20–24% was also observed [69,70]. In addition to the two clinical cases previously mentioned from Germany and Poland [16,17], the presence of piroplasm species primarily associated with dogs, such as *B. vogeli* and *B. canis*, has been sporadically described in cats in Europe [89]. Further details on *Babesia* spp. reported from domestic cats in Europe and worldwide are available in Penzhorn and Oosthuizen [15].

The absence of *Hepatozoon* spp. DNA agrees with the results of a recent survey on cats from northern, central, and southern Italy [71]. Data on the occurrence of this parasite in cats is minimal. In Italy, a single record of *H. felis* was described in 2017 in a cat, out of 330 examined, living on the Aeolian Islands [4]. A subsequent epidemiological study involving 196 cats from three provinces of southern Italy (Bari, Lecce, and Matera) found *Hepatozoon* spp. DNA in 10 cats (5.1%, CI: 3–9%), with the BLAST analysis revealing the presence of three species, *H. canis*, *H. felis*, and *H. silvestris* and, thus, demonstrating for the first time *H. silvestris* infection in a domestic cat [2]. This parasite was described as a novel species in European wild cats from Bosnia and Herzegovina shortly before [90] and subsequently reported as responsible for a fatal infection in a domestic cat from Switzerland [32]. The presence of *H. felis* in cat blood samples has been found, with variable prevalence values, in several European countries, including France (1.7% [52]), Spain (1.6% [27]; 16% [91]), and Portugal (15.6% [89]). Recently, it was described as the cause of a clinical case in Switzerland [92].

## 5. Conclusions

The results of the present study show a widespread presence of ABPs, suggesting that stray cats may represent a potential health threat to companion animals and people and the need for improved management. While these diseases in privately owned cats can be prevented and managed through standard veterinary care, appropriate dewormers, and effective ectoparasiticides, investigating free-roaming, stray, or feral cats may be particularly interesting as they live outdoors, have constant exposure to ticks and fleas, and prey on wildlife that may harbor pathogens. Additionally, stray cats are often neither monitored nor treated for vector-borne pathogens. Considering the obtained results, it would be worthy to conduct further epidemiological surveys investigating other ABPs, responsible for diseases such as rickettsiosis or borreliosis. Knowledge of pathogens in feline populations is helpful for veterinary clinicians in the prevention and control of such agents. Moreover, data about the prevalence of ABPs in feline populations can aid physicians in the diagnosis and control of these zoonotic diseases. Therefore, accurate information on the prevalence of feline pathogens may also be relevant from a One Health perspective.

## Figures and Tables

**Table 1:** Gene target, amplicon length, primer pairs, and PCR conditions used for the different pathogens.

Pathogen	Gene Target and Amplicon Length (bp)	Primers’ Name and Sequence	PCR Conditions	References
*Anaplasma phagocytophilum*	16S rRNA ^1^ 932 bp (First PCR)	GE3a CACATGCAAGTCGAACGGATTATTC GE10r TTCCGTTAAGAAGGATCTAATCTCC	95 °C–30′’ 55 °C–30′’ 72 °C–1′	[58]
	16S rRNA ^1^ 546 bp (Second PCR)	GE9f AACGGATTATTCTTTATAGCTTGCT GE2 GGCAGTATTAAAAGCAGCTCAGGG	95 °C–30′’ 55 °C–30′’ 72 °C–1′	
*Bartonella spp.*	16S rRNA ^1^ 296bp	P24E CCTCCTTCAGTTAGGCTGG P12B GAGATGGCTTTTGGAGATTA	95 °C–1′ 57 °C–1′ 72 °C–1′	[59]
*Coxiella burnetii*	IS1111a ^2^ 687bp	TRANS-1 TATGTATCCACCGTAGCCAGT TRANS-2 CCCAACAACACCTCCTTATTC	95 °C–30′’ 64 °C–1′ 72 °C–1′	[60]
*Ehrlichia canis*	16S rRNA ^1^ 152 bp (First PCR)	ECCf AGAACGAACGCTGGCGGCAAGC ECBr CGTATTACCGCGGCTGCTGGCA	94 °C–1′ 55 °C–2′ 72 °C–1.5′	[61]
	16S rRNA ^1^ 395bp (Second PCR)	ECAN5 CAATTATTTATAGCCTCTGGCTATAGGA HE3r TATAGGTACCGTCATTATCTTCCCTAT	94 °C–1′ 55 °C–2′ 72 °C–1.5′	
*Leishmania* spp.	18S rRNA ^3^ 603 bp (First PCR)	R221 GGTTCCTTTCCTGATTTACG R332 GGCCGGTAAAGGCCGAATAG	94 °C–30′’ 60 °C–30′’ 72 °C–30′’	[62]
	350 bp (Second PCR)	R223 TCCCATCGCAACCTCGCTT R333 AAAGCGGGCGCGGTGCTG	94 °C–30′’ 65 °C–30′’ 72 °C–30′’	
*Babesia/Theileria* spp.	18S rRNA ^3^ 560 bp	Mic1 GTCTTGTAATTGGAATGATGG Mic2 CCAAAGACTTTGATTTCTCTC	94 °C–30′’ 50 °C–30′’ 72 °C–1′	[63]
*Cytauxzoon* spp.	18S rRNA ^3^ 408 bp	Piro-A AATACCCAATCCTGACACAGGG Piro-B TTAAATACGAATGCCCCCAAC	94 °C–30′’ 56 °C–30′’ 72 °C–45′’	[64]
*Hepatozoon* spp.	18S rRNA ^3^ 625 bp	Hep-F ATACATGAGCAAAATCTCAAC Hep-R CTTATTATTCCATGCTGCAG	94 °C–30′’ 57 °C–30′’ 72 °C–1′	[65]

^1^ 16S rRNA: long subunit (16S) ribosomal RNA; ^2^ ISS1111: insertion sequence; ^3^ 18S rRNA: small subunit (18S) ribosomal RNA.

**Table 2:** Results of the serological (indirect immunofluorescent assay, IFA) and molecular (PCR) tests for protozoa and bacteria of the 85 cats analyzed. CI, confidence interval.

Pathogen	IFA		PCR	
	*N* Positive	Seropositivity Rate (%), 95% CI	*N* Positive	Detection Rate (%), 95% CI
*Anaplasma phagocytophilum*	4	4.7, 95% CI 2–9.2	0	0
*Bartonella henselae*	39	45.9, 95% CI 35.3–56.5	23	27.1, 95% CI 17.6–36.5
*Coxiella burnetii*	32	37.6, 95% CI 27.3–47.9	25	29.4, 95% CI 19.7–39.1
*Ehrlichia canis*	12	14.1, 95% CI 6.7–21.5	2	2.4, 95% CI 0–5.6
*Leishmania* spp.	2	2.4, 95% CI 0–5.6	5	5.9, 95% CI 0.9–10.9
*Babesia*/*Theileria* spp.	-	-	0	0
*Cytauxzoon* sp.	-	-	2	2.4, 95% CI 0–5.6
*Hepatozoon* sp.	-	-	0	0

**Table 3:** Details of the cats with coinfections of two or more pathogens. ID, identifier.

Cat ID	Cox Serol	Cox PCR	Bart Serol	Bart PCR	Anapl Serol	Anapl PCR	Ehr Serol	Ehr PCR	Hep PCR	Piro PCR	Cytaux PCR	Leish Serol	Leish PCR
22			pos				pos						
30	pos	pos			pos								
31	pos	pos	pos									pos	pos
38	pos	pos	pos										
40	pos	pos	pos	pos									
44	pos	pos					pos						pos
45	pos												pos
50							pos						pos
52	pos		pos	pos									
54			pos	pos							pos		
62			pos									pos	pos
72			pos	pos			pos						
75			pos	pos							pos		
77					pos		pos						
88	pos	pos					pos						
90	pos	pos	pos	pos									
95			pos	pos			pos						
97	pos	pos	pos	pos									
100	pos	pos	pos	pos									
101	pos		pos	pos									
111	pos		pos	pos									

Cox: *Coxiella burnetii*; Bart: *Bartonella henselae*; Anapl: *Anaplasma phagocytophilum*; Ehr: *Ehrlichia canis*; Hep: *Hepatozoon* spp.; Piro: *Babesia/Theileria* spp.; Cytaux: *Cytauxzoon* spp.; Leish: *Leishmania* spp.
